# Respiratory syncytial virus‐associated acute respiratory illness in adult non‐immunocompromised patients: Outcomes, determinants of outcomes, and the effect of oral ribavirin treatment

**DOI:** 10.1111/irv.12971

**Published:** 2022-02-12

**Authors:** Phunsup Wongsurakiat, Siwadol Sunhapanit, Nisa Muangman

**Affiliations:** ^1^ Division of Respiratory Diseases and Tuberculosis, Department of Medicine, Faculty of Medicine Siriraj Hospital Mahidol University Bangkok Thailand; ^2^ Uttaradit Hospital Tha It Thailand; ^3^ Diagnostic Division, Department of Radiology, Faculty of Medicine Siriraj Hospital Mahidol University Bangkok Thailand

**Keywords:** acute respiratory illness, adult non‐immunocompromised patients, hospital‐free days, mortality, oral ribavirin, outcomes, pneumonia, respiratory syncytial virus

## Abstract

**Background:**

Respiratory syncytial virus (RSV) is an increasingly common cause of respiratory illness in adult non‐immunocompromised patients. Oral ribavirin was reported to improve outcomes of RSV infection in immunocompromised patients. This study aimed to determine the outcomes of non‐immunocompromised patients hospitalized with RSV‐associated acute respiratory illnesses (RSV‐ARI), the factors independently associated with the outcomes and the effect of oral ribavirin treatment.

**Methods:**

This retrospective, observational cohort study included 175 adults admitted to the hospital with virologically confirmed RSV‐ARI during 2014–2019. Severe ARI was identified using Infectious Diseases Society of America/American Thoracic Society (IDSA/ATS) criteria for severe community‐acquired pneumonia. The primary outcome was all‐cause mortality within 30 days after enrollment. A multivariable Cox model was performed to identify significant predictors of mortality.

**Results:**

Mean age was 76 ± 12.7 years. Seventy‐eight (44.6%) patients met the diagnostic criteria for severe ARI. Thirty‐six (20.6%) patients required invasive mechanical ventilation, and 11 (6.3%) required vasopressor. Ninety‐nine (56.6%) patients received oral ribavirin treatment, and 52 (29.7%) received systemic corticosteroids. Forty‐one (23.4%) patients had evidence of bacterial infection. Overall mortality was 7.4%. Mortality among patients with non‐severe ARI and severe ARI was 1.04% and 15.4%, respectively. Estimated glomerular filtration rate <50 ml/min/1.73 m^2^, severe ARI, systemic corticosteroids, and bacterial infection were independently associated with higher risk of mortality. Treatment with oral ribavirin was the only factor associated with reduced mortality (adjusted HR: 0.19, 95% CI: 0.04–0.9, *P* = 0.03).

**Conclusion:**

RSV‐ARI may result in significant mortality and health care utilization. Treatment with oral ribavirin may improve survival in these patients.

**Abbreviations**
aHR: adjusted hazard ratio
ARI: acute respiratory illness
CAP: community‐acquired pneumonia
CI: confidence interval
eGFR: estimated glomerular filtration rate
FiO_2_
: fraction of inspired oxygen
HR: hazard ratio
HAP: hospital‐acquired pneumonia
IDSA/ATS: Infectious Diseases Society of America/American Thoracic Society
IQR: interquartile range
RSV: respiratory syncytial virus
RT‐PCR: reverse transcription polymerase chain reaction
SD: standard deviation
VAP: ventilator‐associated pneumonia

## 1 INTRODUCTION

Respiratory syncytial virus (RSV) is increasingly recognized as a common cause of acute respiratory illness (ARI) in adults.^1^, ^2^, ^3^, ^4^, ^5^ Among adults admitted to the hospital with RSV‐associated acute respiratory illness (RSV‐ARI), 10%–31% require intensive care and 3%–17% need mechanical ventilation.^4^ The reported RSV‐associated morbidity, mortality, and health care utilization were comparable with those observed in patients with influenza infection.^3^, ^6^, ^7^ Antiviral treatment, coexisting bacterial infection, and corticosteroids were reported to significantly influence survival in influenza patients.^8^ However, the relative impact of these factors on the clinical outcomes of patients with RSV‐ARI remains unclear. The antiviral treatment options are limited in RSV infection. Aerosolized ribavirin is approved for the treatment of RSV in pediatric patients. In adult population, the use of ribavirin is off‐label and has been limited to severely immunocompromised transplant recipients. The reported clinical outcomes in those patients were favorable based mainly on observational data.^9^, ^10^ Due to the high cost of aerosolized ribavirin and the occupational risk to healthcare workers exposed to this form of ribavirin, many centers use oral ribavirin instead.^11^ Most studies that have compared oral and inhaled ribavirin have shown similar efficacy.^12^, ^13^, ^14^ However, reports of oral ribavirin's efficacy in adult non‐immunocompromised RSV are comparatively scarce.^15^, ^16^, ^17^ Whether the data that we currently have about the effect of oral ribavirin can be applied to general adult population still needs to be determined. Even though oral ribavirin has not yet been approved for adult use in RSV, it is used to treat adult patients with severe RSV‐ARI at our center. RSV infection is associated with airway inflammation and increased airway responsiveness and may trigger exacerbation of chronic airway diseases. Corticosteroids are widely prescribed for bronchospasm, which is commonly associated with RSV infection.^3^, ^18^

To increase our understanding of RSV‐ARI, this study sets forth to determine the outcomes of patients hospitalized with RSV‐ARI, the factors independently associated with the outcomes, and the effect of oral ribavirin treatment on patient outcomes.

## 2 STUDY DESIGN AND METHODS

We performed a retrospective single‐center study of a cohort of adults hospitalized with RSV‐ARI during January 2014 to April 2019 at a 2300‐bed university‐based national tertiary referral center in Bangkok, Thailand. Patients with RSV infection were identified from our center's inpatient database using the ICD‐10 code related to RSV infection (J12.1: RSV pneumonia; J20.5: Acute bronchitis due to RSV; B97.4: RSV as the cause of diseases classified elsewhere). RSV infection was defined by a positive reverse transcription polymerase chain reaction (RT‐PCR) to any of the following samples: pharyngeal swab, nasal swab, nasopharyngeal aspirate, sputum, or bronchoalveolar lavage (BAL). Patients with RSV infection that met all of the following criteria were included: age ≥18 years; admitted from outside the hospital with signs or symptoms of ARI, defined as two or more respiratory symptoms, including cough, dyspnea, pleuritic chest pain, and/or respiratory distress; and diagnosis of RSV infection within 48 h after admission. Patients who were pregnant, who had received immunosuppressants or long‐term corticosteroid therapy, or who had concomitant acquired immunodeficiency syndrome were excluded.

### 2.1 Data collection

Electronic and written medical records were reviewed for all patients. A protocol for data collection was applied in all cases. Information that was collected included age, gender, comorbidities (pulmonary, cardiovascular, liver, renal, neoplasms, and diabetes), functional status (independent or dependent functional status), time of illness onset, and hospital admission. The following clinical data at admission were recorded: presenting symptoms and signs, mental alterations, heart rate, respiratory rate, and blood pressure. The need for non‐invasive or invasive mechanical ventilation or vasopressors within 48 h of hospital admission was assessed. Chest radiographic findings were also documented (infiltrates, number of lobes affected, unilateral versus bilateral affection, and pleural effusion). The chest radiographs at admission were retrospectively reviewed by three independent physicians (two pulmonologists and one radiologist) with all interpreters blinded to all clinical information and outcomes. The images were then collectively interpreted so that a consensus determination could be reached and documented. The recorded laboratory data included complete blood count, chemical parameters, and arterial blood gas analysis. If the results of arterial blood gas analysis were unavailable, the PaO_2_ was inferred from the oxygen saturation as measured by pulse oximetry (SpO_2_).^19^

All microbiological studies for bacterial infection, including blood cultures, sputum cultures, pleural fluid cultures, or BAL cultures, at admission and during the course of hospitalization were recorded. Etiologic diagnosis was considered positive in the following situations: isolation of a respiratory pathogen in a usually sterile specimen (blood and pleural fluid) or bacterial growth in BAL fluid (≥10^4^ cfu/ml) or a predominant microorganism isolated from a sputum sample (>25 polymorphonuclear leukocytes and <10 squamous cells per low‐power field) with moderate or high quantity.

### 2.2 Medication treatment

Four types of medications prescribed during admission were recorded. **Ribavirin** treatment referred to the administration of ribavirin for one or more doses during the study illness, including timing of initiation and dosing. **Systemic corticosteroids use** was defined as any intended therapeutic use of corticosteroids (parenteral or enteral routes) for the study illness for ≥24 h during hospitalization. Replacement doses and inhalation treatment were excluded. **Inhaled bronchodilator** used to treat the study illness for ≥24 h. **Antibiotic** referred to the initial antibiotic prescribed within the first 24 h.

### 2.3 Definitions

**Severe ARI** was defined according to Infectious Diseases Society of America/American Thoracic Society (IDSA/ATS) criteria for severe community‐acquired pneumonia (CAP)^20^ (Table S1). **Bacterial coinfection** was defined in patients having one or more positive cultures of a known respiratory pathogen thought to be causing a true infection from blood and/or a respiratory sample (sputum, BAL, or pleural fluid) collected within 2 days of admission. **Bacterial superinfection** was defined in patients having one or more positive cultures of a known respiratory pathogen from blood and/or a respiratory sample collected more than 2 days after admission. **Adequate antibiotic therapy** was defined as treatment with at least one agent to which all recovered isolates were susceptible in vitro. **Non‐respiratory nosocomial infection** was defined in patients having one or more positive cultures with a bacterial pathogen thought to be causing a true infection from non‐respiratory sources collected more than 2 days after admission.

### 2.4 Outcomes

The primary outcome was all‐cause mortality within 30 days after admission. The secondary outcome was the duration of hospitalization by assessing the number of days alive and outside the hospital (hospital‐free days) within 30 days after hospital admission.

### 2.5 Statistical analysis

Descriptive analysis was performed. Discrete variables are expressed as number and percentage (%) and continuous variables as either mean ± deviation (SD) or median and interquartile range (IQR). Proportions were compared using chi‐square test or Fisher's exact test for categorical variables and nonparametric Mann–Whitney *U* test or unpaired *t* test for continuous variables.

Factors affecting patient survival were analyzed. Time to in‐hospital death was defined as the time from hospital admission to date of death within 30 days after admission. Data were censored at either hospital discharge or the date of the final daily record for patients alive and still in the hospital or at 30 days from admission, whichever was first. Univariate and multivariate Cox regression analyses were performed to determine predictive factors of mortality by estimating crude hazard ratio (HR) and adjusted hazard ratio (aHR) and their 95% confidence intervals (CIs). Factors with a *P* value <0.20 in univariate analysis, biologically relevant potential clinical confounders (age, comorbidities, and functional status), and ribavirin treatment were entered into backward stepwise multiple variable Cox regression model to identify factors independently associated with hospital mortality. A lower HR (HR < 1) indicates a lower probability of death.

Factors affecting duration of hospitalization were analyzed using time‐to‐event analyses. Univariate and multivariate Cox regression analyses were performed to identify factors independently associated with the time to hospital discharge alive within 30 days after admission, with patients who were not discharged at day 30 or death before day 30 were considered as right censored at day 30. A lower HR (HR < 1) indicates a lower probability of hospital discharge alive and therefore increased duration of hospitalization. Kaplan–Meier curves were used to illustrate time to hospital death and time to hospital discharge alive. All statistical analyses were two‐sided, and a *P* value <0.05 was considered to be statistically significant. All statistical analyses were performed using SPSS Statistics software version 20 (SPSS, Inc., Chicago, IL, USA).

## 3 RESULTS

**FIGURE 1:**
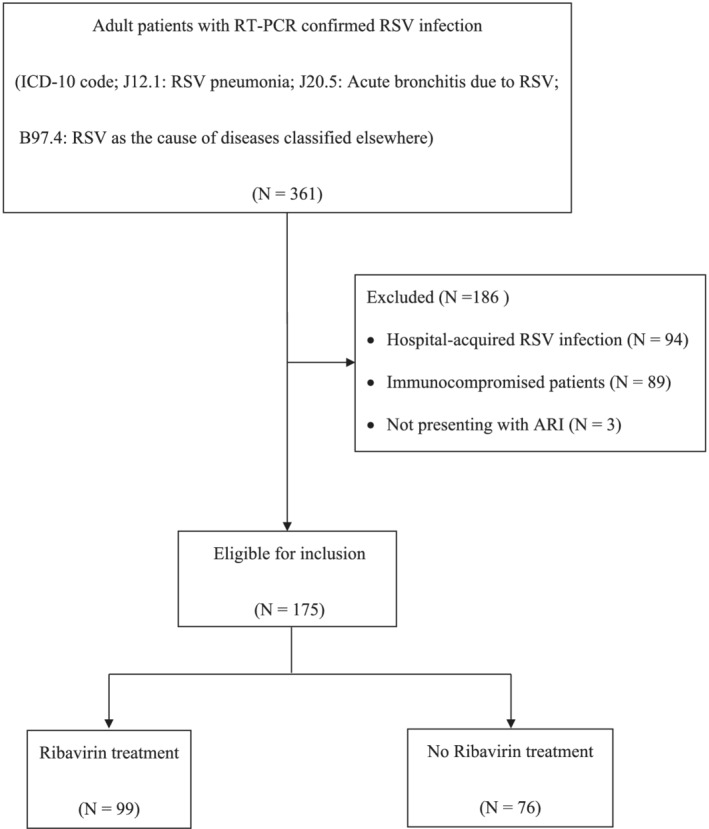
Flow chart of included patients with community‐acquired RSV‐associated ARI. ARI, acute respiratory illness; RSV, respiratory syncitial virus

**FIGURE 2:**
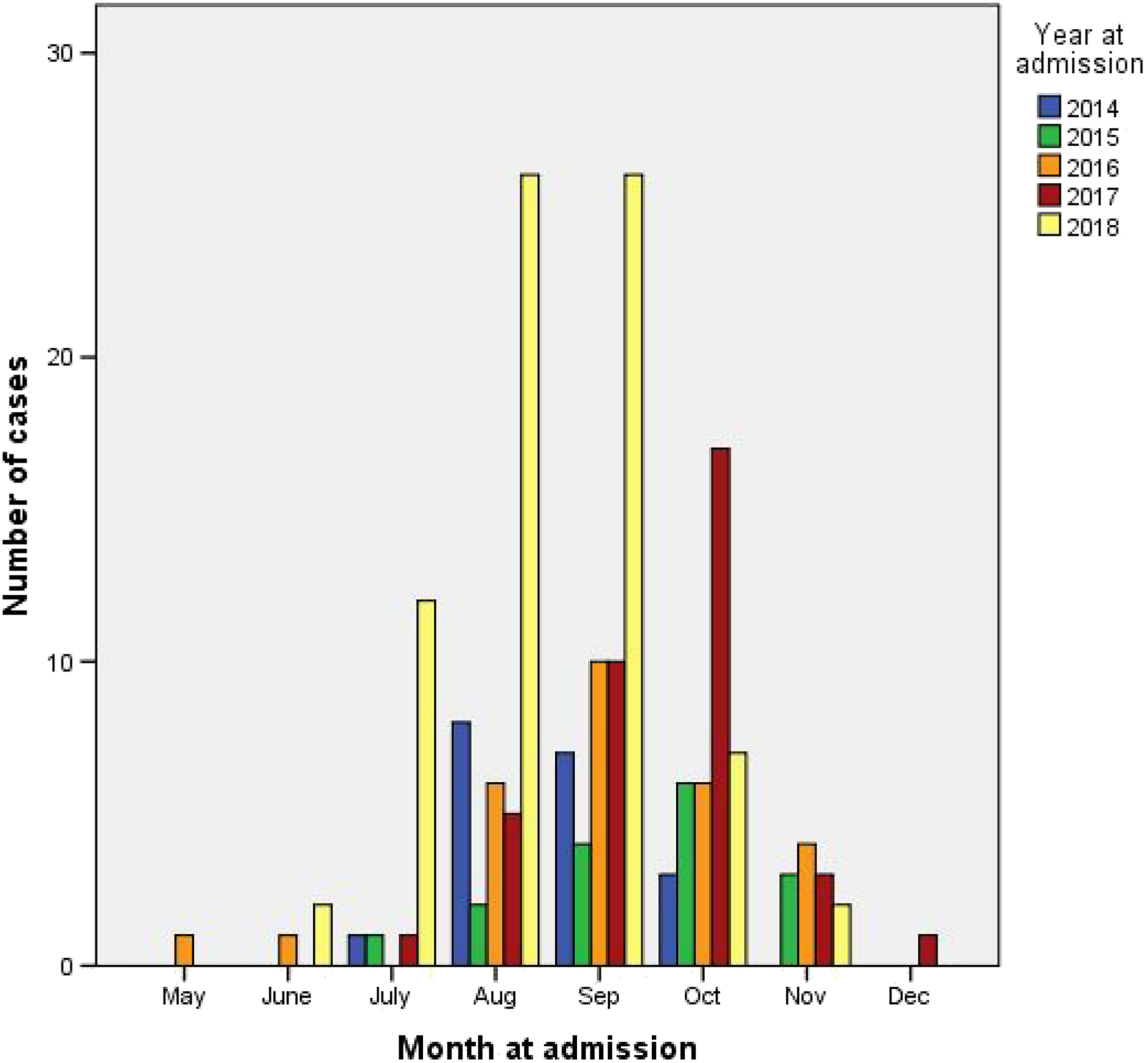
Monthly distribution of respiratory syncytial virus infections in this study

A total of 175 adult patients with community‐acquired RSV‐ARI were included in this study (Figure 1). All cases occurred during the rainy and winter season in Thailand (Figure 2). The mean age was 76 ± 12.7 years, and 165 (94.3%) patients had one or more coexisting medical conditions. One hundred and fifty‐nine (90.6%) patients had infiltrates on chest radiograph (Table 1). Seventy‐eight (44.6%) patients met the diagnostic criteria for severe ARI. Thirty‐six (20.6%) patients required invasive mechanical ventilation, 11 (6.3%) required vasopressor, and 56 (32%) were classified as severe ARI by IDSA/ATS minor criteria for severe CAP. Seven (4%) patients required invasive mechanical ventilation later during the course of hospitalization. Clinical outcomes according to ARI severity are shown in Table 2.

**TABLE 1:** Demographic, clinical characteristics, and mortality at 30 days of all patients hospitalized with respiratory syncytial virus‐associated acute respiratory illness

	Alive (*N* = 162)	Dead (*N* = 13)	*P* value
Year at admission:			0.1
2014	15 (9.3)	4 (30.8)	
2015	15 (9.3)	1 (7.7)	
2016	28 (17.3)	0	
2017	34 (21)	3 (23.1)	
2018	70 (43.2)	5 (38.5)	
Month at admission:			0.24
May	1 (0.6)	0	
June	2 (1.2)	1 (7.7)	
July	15 (9.3)	0	
August	41 (25.3)	6 (46.1)	
September	55 (33.9)	2 (15.4)	
October	35 (21.6)	4 (30.8)	
November	12 (7)	0	
December	1 (0.6)	0	
Age, year	75.9 ± 12.7	76.8 ± 11.2	0.81
Gender (male)	64 (39.5)	3 (23.1)	0.37
Comorbid:			
Cardiovascular diseases	136 (83.9)	10 (76.9)	0.7
Diabetes	61 (37.6)	3 (23.1)	0.38
Chronic kidney diseases	81 (50)	8 (61.5)	0.42
Chronic lung diseases	45 (27.8)	5 (38.5)	0.52
Malignant diseases	15 (9.6)	1 (7.7)	0.1
Number of comorbid conditions:			0.82
≤1	49 (30.2)	5 (38.5)	
2	49 (30.2)	3 (23.1)	
≥3	64 (39.5)	5 (38.5)	
Dependent functional status	49 (30.2)	3 (23.1)	0.76
eGFR^a^, ml/min/1.73 m^2^ eGFR^a^ < 50 ml/min/1.73 m^2^	56.3 ± 30.4 73 (45.1)	41.4 ± 22.4 10 (76.9)	0.04^*^ 0.03^*^
Chest radiograph:			
Infiltrates	146 (90.1)	13 (100)	0.38
Bilateral infiltrates	61 (37.6)	6 (46.1)	0.56
Multilobar infiltrates	77 (47.5)	11 (84.6)	0.01^*^
PaO_2_/FiO_2_, median (IQR), mmHg	236 (196–273)	273 (177–514)	0.16
Hemolobin, g/dl	11.6 ± 2.2	10.7 ± 3.2	0.17
WBC count, cells × 10^3^/mm^3^	9.5 ± 4.3	10 ± 3.9	0.67
Absolute lymphocyte count, median (IQR), cells × 10^3^/mm^3^	1.3 (0.8–1.9)	1.6 (0.7–2.3)	0.46
Absolute lymphocyte count < 0.8 cells × 10^3^/mm^3^	40 (24.7)	4 (30.8)	0.74

*Note*: Data are presented as mean ± SD or *n* (%), unless otherwise stated.

Abbreviation: IQR, interquartile range.

^a^ Glomerular filtration rate estimated by CKD‐EPI creatinine equation.

^*^ Statistically significant difference.

**TABLE 2:** Severity, treatment and mortality at 30 days of all patients hospitalized with respiratory syncytial virus‐associated acute respiratory illness

	Alive (*N* = 162)	Dead (*N* = 13)	*P* value
ICU admission	12 (7)	2 (15)	0.6
Invasive mechanical ventilation	26 (16)	10 (76.9)	<0.001*
Vasopressor requirement	8 (4.9)	3 (23.1)	0.04*
Minor criteria ≥3^a^:	51 (31.5)	5 (38.5)	0.76
Confusion/disorientation	19 (11.7)	3 (23.1)	0.38
Hpotension	7 (4.3)	0	0.66
Nonivasive ventilation	20 (12.3)	1 (7.7)	0.71
PaO_2_/FiO_2_ ≤ 250 mmHg	88 (54.3)	5 (38.5)	0.27
Multilobar infiltrates	77 (47.5)	11 (84.6)	0.01*
BUN ≥20 mg/dl	67 (41.3)	7 (53.8)	0.38
WBC count <4000 cells/mm^3^	9 (5.6)	1 (7.7)	1
Platelet count <100 000 cells/mm^3^	12 (7)	2 (15)	0.6
Severe acute respiratory illness^b^	66 (40.7)	12 (92.3)	<0.001*
Bacterial coinfection	25 (15.4)	5 (38.5)	0.05*
Bacterial superinfection:	11 (6.8)	7 (53.8)	<0.001*
Hospital‐acquired pneumonia	2 (1.2)	0	
Ventilated hospital‐acquired pneumonia	5 (3.1)	2 (15.4)	
Ventilator‐associated pneumonia	4 (2.5)	5 (38.5)	
Bacterial coinfection and bacterial superinfection	4 (2.5)	3 (23.1)	0.001*
Bacterial infection^c^	32 (19.7)	9 (69.2)	<0.001*
Positive blood culture	2/123 (1.6)	1/12 (8.3)	0.24
Initial antibiotic treatment	137 (84.6)	13 (100)	0.22
Inadequate initial antibiotic treatment^d^	5/25 (20)	2/5 (40)	0.56
Non‐respiratory nosocomial infection	11 (6.8)	1 (7.7)	1
Ribavirin treatment	90 (55.6)	9 (69.2)	0.34
Systemic corticosteroids use	44 (27.2)	8 (61.5)	0.01*
Bronchodilator therapy	145 (89.5)	12 (92.3)	1
Length of stay in hospital, median (IQR), *d*	9 (6–15)	10 (8.5–21.5)	0.21
Hospital‐free days^e^, median (IQR), *d*	21 (15–24)	0	<0.001*

*Note*: Data are presented as mean ± SD or *n* (%), unless otherwise stated.

Abbreviation: IQR, interquartile range.

^a^ IDSA/ATS minor criteria for severe community‐acquired pneumonia.^20^

^b^ Defined by IDSA/ATS criteria for severe community‐acquired pneumonia.^20^

^c^ Bacterial coinfection and/or superinfection.

^d^ Pathogens detected were not susceptible to the antibiotics administered within 24 h of presentation.

^e^ Number of days from admission to day 30 that the patient was not admitted to the hospital.

^*^ Statistically significant difference.

### 3.1 Medication treatment

Ninety‐nine (56.6%) patients received treatment with oral ribavirin. Of those, 64 (64.6%) received treatment within 24 h after admission, and 89 (89.9%) received treatment within 48 h after admission. The most common dosing regimen prescribed was 600‐mg loading, followed by 200 mg three times a day for 7 days. Patients who were likely to receive treatment with ribavirin were patients with chronic lung diseases, severe ARI, and patients who had infiltrates on chest radiograph (Table 3). There was no document of serious adverse event related to ribavirin leading to cessation of treatment. Compared with patients who did not receive ribavirin, patients who received ribavirin tended to have lower hemoglobin level during hospitalization. Nevertheless, there was no significant difference in blood transfusion requirement and the change of white blood cell count (Table 3). Fifty‐two (29.7%) patients received treatment with systemic corticosteroids, and 157 (89.7%) patients received inhaled bronchodilator therapy during hospitalization. Patients who received treatment with ribavirin were more likely to receive treatment with systemic corticosteroids or inhaled bronchodilator (Table 3). The clinical outcomes associated with these medication treatments are shown in Table 2.

**TABLE 3:** Demographic, clinical characteristics, and clinical outcomes of all patients by ribavirin treatment

	No ribavirin treatment (*N* = 76)	Ribavirin treatment (*N* = 99)	*P* value
Year at admission:			<0.001*
2014	9 (11.8)	10 (10.1)	
2015	11 (14.5)	5 (5)	
2016	16 (21)	12 (12.1)	
2017	22 (28.9)	15 (15.1)	
2018	18 (23.7)	57 (57.6)	
Month at admission:			0.23
May	0	1 (1)	
June	0	3 (3)	
July	7 (9.2)	8 (8.1)	
August	16 (21)	31 (31.3)	
September	24 (31.6)	33 (33.3)	
October	21 (27.6)	18 (18.2)	
November	7 (9.2)	5 (5)	
December	1 (1.3)	0	
Age, year	74.2 ± 12.7	77.3 ± 12.3	0.11
Comorbid:			
Cardiovascular diseases	62 (81.6)	84 (84.8)	0.56
Diabetes	27 (35.5)	37 (37.4)	0.8
Chronic kidney diseases	39 (51.3)	50 (50.5)	0.91
Chronic lung diseases	15 (19.7)	35 (35.3)	0.02*
Malignant diseases	9 (11.8)	7 (7.1)	0.28
Number of comorbid conditions:			0.15
≤1	23 (30.3)	31 (31.3)	
2	28 (36.8)	24 (24.2)	
≥3	25 (32.9)	44 (44.4)	
Dependent functional status	24 (31.6)	28 (28.3)	0.64
Infiltrates on chest radiograph	64 (84.2)	95 (96)	0.008*
Invasive mechanical ventilation	8 (10.5)	28 28.3)	0.004*
Vasopressor requirement	5 (6.6)	6 (6.1)	1
Minor criteria ≥3^a^:	19 (25)	37 (37.4)	0.08
Confusion/disorientation	11 (14.5)	11 (11.1)	0.51
Hpotension	4 (5.3)	3 (3)	0.7
Non‐invasive ventilation	9 (11.8)	12 (12.1)	0.95
PaO_2_/FiO_2_ ≤ 250 mmHg	39 (51.3)	54 (54.5)	0.67
Multilobar infiltrates	28 (36.8)	60 (60.6)	0.002*
BUN ≥20 mg/dl	27 (35.5)	47 (47.5)	0.11
WBC count <4000 cells/mm^3^	2 (2.6)	8 (8.1)	0.19
Platelet count <100 000 cells/mm^3^	4 (5.3)	10 (10.1)	0.24
Severe acute respiratory illness^b^	24 (31.6)	54 (54.5)	0.002*
ICU admission	3 (3.9)	11 (11.1)	0.08
Systemic corticosteroids use	16 (21)	36 (36.4)	0.03*
Bronchodilator therapy	64 (84.2)	93 (93.9)	0.04*
Bacterial coinfection	10 (13.2)	20 (20.2)	0.22
Bacterial superinfection	2 (2.6)	16 (16.2)	0.003*
Bacterial infection^c^	11 (14.5)	30 (30.3)	0.01*
Non‐respiratory nosocomial infection	4 (5.3)	8 (8.1)	0.46
Mortality at 30 days	4 (5.3)	9 (9.1)	0.34
Length of stay in hospital, median (IQR), *d*	7 (5–11)	11 (8–17)	<0.001*
Hospital‐free days^d^, median (IQR), *d*	22.5 (17.2–25)	18 (9–22)	<0.001*
Baseline hemolobin, g/dl	11.7 ± 2.2	11.4 ± 2.4	0.48
Lowest hemoglobin during hospitalization, g/dl	10.8 ± 2.3	10 ± 2.5	0.045*
Difference between baseline hemoglobin and lowest hemoglobin, median (IQR), g/dl	1 (0–1)	1 (0–2)	0.1
Blood transfusion requirement	6 (7.9)	16 (16.2)	0.1
Baseline WBC count, cells × 10^3^/mm^3^	9.8 ± 3.9	9.3 ± 4.6	0.47
Lowest WBC count during hospitalization, median (IQR), cells × 10^3^/mm^3^	7.2 (5.4–9.4)	6.4 (4.6–8.8)	0.13
Difference between baseline WBC count and lowest WBC count, median (IQR), cells × 10^3^/mm^3^	0.49 (0–2.3)	0.93 (0–2.9)	0.43

*Note*: Data are presented as mean ± SD or *n* (%), unless otherwise stated.

Abbreviation: IQR, interquartile range.

^a^ IDSA/ATS minor criteria for severe community‐acquired pneumonia.^20^

^b^ Defined by IDSA/ATS criteria for severe community‐acquired pneumonia.^20^

^c^ Bacterial coinfection and/or superinfection.

^d^ Number of days from admission to day 30 that the patient was not admitted to the hospital.

^*^ Statistically significant difference.

### 3.2 Bacterial infection

**FIGURE 3:**
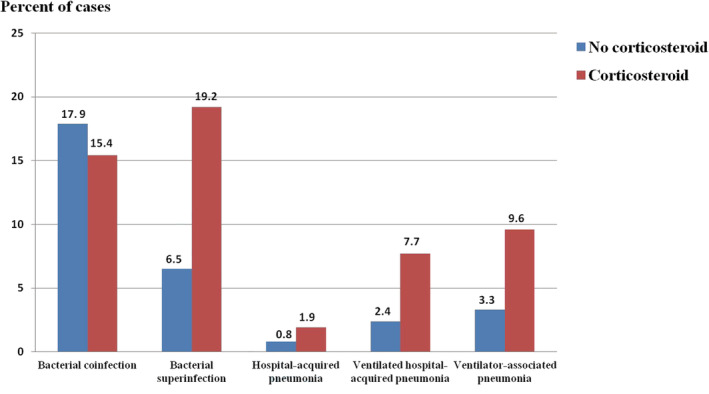
Prevalence of bacterial coinfection and bacterial superinfection according to systemic corticosteroids use. Bacterial superinfection occurred more frequently in patients receiving corticosteroids (19.2% vs. 6.5%, *P* = 0.01)

Forty‐one (23.4%) patients had evidence of bacterial infection during hospitalization. Thirty (17.1%) patients had bacterial coinfection, and 18 (10.3%) had bacterial superinfection (Table 2). Bacterial superinfection occurred more frequently in patients receiving corticosteroids (19.2% vs. 6.5%, *P* = 0.01) (Figure 3). Initial antibiotic treatment was prescribed in 150 (85.7%) patients. Twelve (6.9%) patients developed non‐respiratory nosocomial infection during hospitalization. The clinical outcomes associated with bacterial infection are shown in Table 2.

### 3.3 Outcomes

**FIGURE 4:**
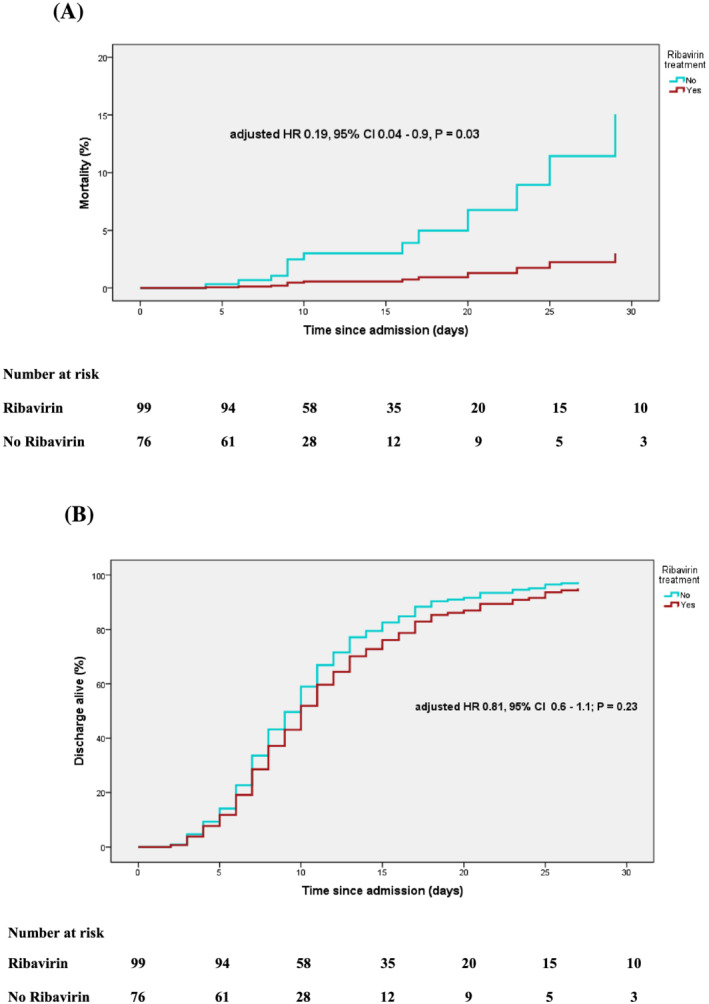
Kaplan–Meier estimates for overall mortality (A) and discharge alive from hospital (B) within 30 days after admission for patients receiving ribavirin treatment and those not receiving ribavirin treatment. The adjusted hazard ratio (aHR) is provided with the 95% CI and *P* value from the multivariate Cox regression model (for details, see Table 4)

Overall mortality within 30 days was 7.4%. Mortality among patients with non‐severe ARI and severe ARI was 1.04% and 15.4%, respectively. Multivariate Cox regression analysis revealed estimated glomerular filtration rate (eGFR) < 50 ml/min/1.73 m^2^, severe ARI, systemic corticosteroids, and bacterial infection to be independent predictors of 30‐day mortality. Ribavirin treatment was the only factor independently associated with lower risk of mortality (Table 4 and Figure 4).

**TABLE 4:** Univariate and multivariate Cox regression analyses of potential factors affecting mortality and time to hospital discharge alive within 30 days after admission

Factor affecting mortality^a^	Univariate model			Multivariate model		
	Hazard ratio	95% CI	*P* value	Hazard ratio	95% CI	*P* value
Age	0.99	0.95–1	0.78	‐	‐	‐
Number of comorbid conditions ≤1	1			‐	‐	‐
Number of comorbid conditions = 2	0.59	0.1–2.5	0.47	‐	‐	‐
Number of comorbid conditions >3	0.74	0.2–2.6	0.63	‐	‐	‐
Dependent functional status	0.55	0.1–2	0.36	‐	‐	‐
Ribavirin treatment	1	0.31–3.3	0.99	0.19	0.04–0.9	0.03*
eGFR <50 ml/min/1.73 m ^2^ ^b^	2.7	0.7–10	0.13	4.4	1.1–18.4	0.04*
Severe acute respiratory illness^c^	6.4	0.8–49.8	0.08	8.5	1–69.9	0.046*
Systemic corticosteroids use	3.2	1–9.8	0.04*	6.3	1.7–23.9	0.007*
Bacterial infection^d^	3.5	1–11.6	0.04*	5.5	1.4–20.6	0.01*

Factor affecting time to hospital discharge alive^e^						
Age	0.99	0.98–1	0.12	‐	‐	‐
Number of comorbid conditions ≤1	1					
Number of corbid conditions = 2	1	0.7–1.5	0.99	‐	‐	‐
Number of corbid conditions ≥3	0.97	0.7–1.4	0.88	‐	‐	‐
Dependent functional status	0.83	0.6–1.2	0.29	‐	‐	‐
ICU admission	0.47	0.2–0.9	0.02*	‐	‐	‐
Infiltrates on chest radiograph	0.59	0.4–0.9	0.01*	‐	‐	‐
Initial antibiotic treatment	0.46	0.3–0.6	<0.001*	‐	‐	‐
Ribavirin treatment	0.57	0.4–0.8	0.001*	0.81	0.6–1.1	0.23
eGFR <50 ml/min/1.73 m ^2^ ^b^	0.68	0.5–0.9	0.02*	‐	‐	‐
Severe acute respiratory illness^c^	0.36	0.3–0.5	<0.001*	0.43	0.3–0.6	<0.001*
Systemic corticosteroids use	0.73	0.5–1	0.09	0.68	0.5–1	0.048*
Bacterial infection^d^	0.37	0.2–0.6	<0.001*	0.41	0.3–0.6	<0.001*
Hemoglobin	1.1	1–1.2	0.02*	1.1	1–1.2	0.007*
Bronchodilator use	0.5	0.3–0.8	0.01*	0.47	0.3–0.8	0.01*
Non‐respiratory nosocomial infection	0.33	0.2–0.7	0.003*	0.3	0.1–0.6	0.001*

Abbreviations: CI, confidence interval; eGFR, estimated glomerular filtration rate.

^a^ Factor affecting mortality, a hazard ratio <1 indicated a lower probability of death.

^b^ Glomerular filtration rate estimated by CKD‐EPI creatinine equation.

^c^ Defined by IDSA/ATS criteria for severe community‐acquired pneumonia.^20^

^d^ Bacterial coinfection and/or superinfection.

^e^ Factor affecting time to hospital discharge alive, a hazard ratio <1 indicated a lower probability of hospital discharge alive within 30 days after admission.

^*^ Statistically significant difference.

The average length of hospital stay was 9 (IQR: 6–15 days). Patient demographic data and clinical characteristics and associated hospital‐free days are shown in Tables S2–S4. Multivariate analysis revealed severe ARI, systemic corticosteroids, bacterial infection, lower hemoglobin, bronchodilator use, and non‐respiratory nosocomial infection to be independently associated with longer time to hospital discharge alive within 30 days after admission (Table 4 and Figure 4).

## 4 DISCUSSION

We found the RSV‐ARI patients to be older adults with many coexisting medical conditions. The mortality rate was 7.4%, which is comparable with previously reported mortality rates of 6%–13% in patients hospitalized with RSV infection.^3^, ^4^, ^6^, ^7^, ^21^, ^22^, ^23^ We also found that treating these infections requires substantial health care utilization. The average length of stay was 9 days, and approximately 25% of patients required invasive mechanical ventilation.

Disease severity at admission, low eGFR, bacterial infection, and systemic corticosteroids use were associated with increased mortality. Treatment with oral ribavirin was the only factor associated with reduced mortality. Factors previously reported to be associated with mortality in patients hospitalized with RSV infection were advanced age,^6^ chronic cardiopulmonary disease,^2^, ^23^ multiple prior hospitalizations,^22^ lower respiratory tract infection or pneumonia,^6^, ^22^, ^23^ very severe tachypnea,^22^ altered level of consciousness,^22^, ^23^ elevated blood urea level,^6^, ^23^ need for ventilatory support,^6^ and bacterial coinfection.^6^, ^24^, ^25^, ^26^

There is currently no scoring system specifically designed to assess the severity of patients hospitalized with RSV infection. We used IDSA/ATS criteria for severe CAP to define severe ARI due to its good performance for identifying patients at risk for deterioration or escalation of care, ICU admission, and mortality.^20^ Moreover, these criteria are based mainly on severity of pneumonia rather than other factors, such as age or comorbidities, and they are simple to apply. The common clinical diagnoses of hospitalized RSV infection include pneumonia, exacerbation of chronic airway diseases, and heart failure, of which almost half of patients presented with a clinical diagnosis of pneumonia.^3^, ^6^, ^7^ Pneumonia should be suspected in patients with systemic signs of infection, clinical ARI, and evidence of parenchymal infiltrate on chest radiograph. Patients with RSV‐ARI had RSV infection with clinical ARI. Given the clinical overlapping of these diagnoses and the fact that chest radiograph lacks sensitivity and specificity,^27^ it is reasonable to assume that these patients might have pneumonia or pneumonia combined with other conditions, so assessment and management via the clinical pathway based on assumed pneumonia would be appropriate. Furthermore, 90% of patients in this study had infiltrates on chest radiograph. This criterion could effectively stratify patients with RSV‐ARI as non‐severe ARI with mortality of only 1.04%, or severe ARI with much higher mortality of 15.4%, and a longer duration of hospitalization.

Bacterial coinfection was reported in 9%–30% of patients hospitalized with RSV infection.^6^, ^24^, ^25^, ^26^ The present study documented bacterial coinfection in 17.1% of cases. However, the bacterial testing was neither comprehensive nor systematic and was left to the discretion of the attending physician leading to underestimates of bacterial infection. The true incidence could be even higher since only culture‐proven cases were reported. Moreover, 10.3% of patients developed respiratory bacterial superinfection later during the course of hospitalization. Most cases were ventilated HAP or VAP, which are severe hospital‐acquired infections associated with high mortality. Bacterial infection was found to be independently associated with both increased mortality and extended period of hospitalization. It is, therefore, recommended that patients infected with RSV be carefully evaluated and treated for possible bacterial coinfection. In addition, measures to prevent nosocomial infections, particularly VAP, should be implemented.

RSV infection is associated with airway inflammation and increased airway responsiveness and may trigger exacerbation of chronic airway diseases. Wheezing was reported in 33%–90% of patients with RSV infection.^18^ Almost 90% of patients in this study received inhaled bronchodilator therapy during hospitalization. Systemic corticosteroids are widely used to decrease airway inflammation and obstruction.^3^, ^18^, ^28^ One‐third of patients in this cohort received treatment with systemic corticosteroids, which were found to be independently associated with a sixfold increase in mortality and an extended duration of hospitalization. Humoral immunity may be diminished with the use of systemic corticosteroids.^28^ We found more superinfections among patients receiving systemic corticosteroids. A retrospective study in adult allogeneic stem cell transplant recipients demonstrated that adjunct corticosteroid use in the setting of RSV infection led to higher risk of disease progression.^29^ Similarly, systemic corticosteroid use was associated with longer hospitalization and more secondary infections of adults hospitalized for RSV infections.^6^ Taken together, our results suggest that systemic corticosteroids should be avoided in patients with RSV infection. In patients with established indications, such as therapy for acute airway disease exacerbations, alternative treatment such as high‐dose inhaled corticosteroids should be considered.^30^

Almost half of patients had an eGFR less than 50 ml/min/1.73 m^2^ and was found to be an independent predictor of mortality, with a fourfold increased risk of death. Few retrospective studies reported that elevated blood urea level was associated with increase mortality of patients hospitalized with RSV infection.^6^, ^23^ It is unclear whether preexisting chronic kidney disease or direct renal injury by RSV infection or both led to this finding. An animal model demonstrated that RSV could aggravate renal injury via cytokine release and direct renal injury.^31^

Currently, the standard of care for the management of RSV infection in adults is mainly limited to supportive care. To our knowledge, the present study is the first to evaluate the effect of oral ribavirin in non‐immunocompromised adults with RSV‐ARI. Oral ribavirin treatment was associated with a reduction in mortality of non‐immunocompromised adults with RSV‐ARI. Ribavirin had no effect in the univariate model. This may be explained by indication bias characterized by physicians treating more severe patients with ribavirin. Nevertheless, ribavirin therapy had no effect on duration of hospitalization. The reason for this is suggested to be a consequence of improved survival of more severe patients as a result of their being treated with ribavirin. Similar to the findings in other studies, oral ribavirin appears to be a safe and well‐tolerated treatment.^12^, ^13^, ^14^

The strengths of this study include a homogeneous cohort of adult, non‐immunocompromised patients with community‐acquired RSV infection with virological confirmation by RT‐PCR. A large percentage (56.6%) of patients receiving ribavirin led to well powered to detect differences in treatment efficacy. Moreover, important confounders were carefully adjusted for in multivariate analysis, and there was no missing outcome‐related data.

This study has some mentionable limitations. First, the study is a retrospective single‐center study which is vulnerable to biases and incomplete data. Second, although we took many possible confounding factors into account by adjusting for them, residual confounding cannot be completely excluded. Our cohort comprised mostly older adults with many coexisting medical conditions, so our outcomes might be confounded by different levels of unmeasured patient preference for life‐sustaining treatment. Third, initiation for ribavirin treatment could be subject to indication bias since it was not always clear why some patients were given ribavirin and some were not. Furthermore, standardized dosing and duration of ribavirin has not been established in adults, so dosing and duration were decided according to the discretion of the attending physician. Fourth, the reporting of adverse events during ribavirin treatment may be inaccurate.

## 5 CONCLUSION

RSV‐ARI in adult non‐immunocompromised patients may result in significant mortality and health care utilization. Estimated GFR < 50 ml/min/1.73 m^2^, severe ARI, systemic corticosteroids, and bacterial infection were identified as independent predictors of mortality, and oral ribavirin treatment may improve survival in these patients. RSV infection in patients hospitalized with ARI should be recognized, and treatment with oral ribavirin should be considered, especially in patients with severe ARI. Randomized controlled trials are warranted to validate our findings.

## AUTHOR CONTRIBUTIONS

**Phunsup Wongsurakiat:** Conceptualization; data curation; formal analysis; investigation; methodology; project administration; resources; software; supervision; validation; visualization. **Siwadol Sunhapanit:** Conceptualization; data curation; formal analysis; investigation; methodology; project administration; resources; software; supervision; validation; visualization. **Nisa Muangman:** Conceptualization; data curation; formal analysis; investigation; methodology; project administration; resources; software; supervision; validation; visualization.

## FUNDING INFORMATION

This research received no specific grants from any funding agency in the public, commercial, or non‐profit sectors.

### PEER REVIEW

The peer review history for this article is available at https://publons.com/publon/10.1111/irv.12971.

## Supporting information


**Table S1** Infectious Diseases Society of America/American Thoracic Society criteria for defining severe community‐acquired pneumonia
**Table S2** Demographic, clinical characteristics, and hospital‐free days within 30 days of all patients hospitalized with respiratory syncytial virus‐associated acute respiratory illness
**Table S3** Severity at admission and hospital‐free days within 30 days of all patients hospitalized with respiratory syncytial virus‐associated acute respiratory illness
**Table S4** Bacterial coinfection, bacterial superinfection, treatment and hospital‐free days within 30 days of all patients hospitalized with respiratory syncytial virus‐associated acute respiratory illness

## Data Availability

The data that support the findings of this study are available from the corresponding author upon reasonable request.
