# Supplementary material for: Respiratory syncytial virus‐associated acute respiratory illness in adult non‐immunocompromised patients: Outcomes, determinants of outcomes, and the effect of oral ribavirin treatment
Source: Influenza Other Respir Viruses. 2022 Feb 12;16(4):767–79. doi: 10.1111/irv.12971 (PMC9178057; doi:10.1111/irv.12971)
Supplement: Supplementary file 1 — Table S1 Infectious Diseases Society of America/American Thoracic Society criteria for defining severe community‐acquired pneumonia Table S2 Demographic, clinical characteristics, and hospital‐free days within 30 days of all patients hospitalized with respiratory syncytial virus‐associated acute respiratory illness Table S3 Severity at admission and hospital‐free days within 30 days of all patients hospitalized with respiratory syncytial virus‐associated acute respiratory illness Table S4 Bacterial coinfection, bacterial superinfection, treatment and hospital‐free days within 30 days of all patients hospitalized with respiratory syncytial virus‐associated acute respiratory illness [file IRV-16-767-s001.docx]

**SUPPORTING INFORMATION**

**Respiratory syncytial virus-associated acute respiratory illness in adult non-immunocompromised patients: outcomes, determinants of outcomes and the effect of oral ribavirin treatment**

Phunsup Wongsurakiat^a^, MD, Siwadol Sunhapanit^b^, MD, Nisa Muangman^c^, MD

^a^Division of Respiratory Diseases and Tuberculosis, Department of Medicine, Faculty of Medicine Siriraj Hospital, Mahidol University, 2 Wanglang Road, Bangkoknoi, Bangkok 10700, Thailand. *phunsup.won@mahidol.ac.th*

^b^Uttaradit Hospital, 38 Chetsada Bodin Road, Tha It, Mueang Uttaradit 53000, Thailand. siwadol2530@gmail.com.

^c^Diagnostic Division, Department of Radiology, Faculty of Medicine Siriraj Hospital, Mahidol University, 2 Wanglang Road, Bangkoknoi, Bangkok 10700, Thailand. mailtonisa@gmail.com

**Table S1** Infectious Diseases Society of America/American Thoracic Society criteria for defining severe community-acquired pneumonia [1]

| **Major criteria**   - Respiratory failure requiring invasive mechanical ventilation - Septic shock with need for vasopressors | **Minor criteria**   - Confusion/disorientation - Hypotension requiring aggressive fluid resuscitation - Hypothermia (core temperature < 36°C) - Respiratory rate > 30 breaths/min or need for non-invasive ventilation - Multilobar infiltrates - PaO_2_/FiO_2_ ratio < 250 mmHg - Blood urea nitrogen level > 20 mg/dL) - Leukopenia (white blood cell count < 4,000 cells/mm^3^) - Thrombocytopenia (platelet count < 100,000 cells/mm^3^) |
| --- | --- |
| **Definition includes > 1 major criterion or > 3 minor criteria** | |

**Table S2** Demographic, clinical characteristics, and hospital free days within 30 days of all patients hospitalized with respiratory syncytial virus-associated acute respiratory illness

|  | Hospital-free days within 30 days^a^,  median (IQR), days | *P* value |
| --- | --- | --- |
| Year at admission:  2014 (N = 19)  2015 (N = 16)  2016 (N = 28)  2017 (N = 37)  2018 (N = 75) | 14 (0 - 19)  22.5 (15.2 - 24)  20 (17 - 23)  21 (12.5 - 25)  21 (13 - 23) | .07 |
| Month at admission:  May (N = 1)  June (N = 3)  July (N = 15)  August (N = 47)  September (N = 57)  October (N = 39)  November (N = 12)  December (N = 1) | 12  0 (0 - 10.5)  23 (5 - 24)  20 (4 - 23)  19 (13.5 - 23)  22 (17 - 25)  23 (17 - 24)  16 | .18 |
| Age:  > 65 y (N = 146)  < 65 y (N = 29) | 20 (11.7 - 23)  23 (19 - 24.5) | .05* |
| Gender:  Male (N = 67)  Female (N = 108) | 21 (13 - 24)  20 (12.2 - 23) | .2 |
| Comorbid:  Cardiovascular diseases  Yes (N = 146)  No (N = 29)  Diabetes  Yes (N = 64)  No (N = 111)  Chronic kidney diseases  Yes (N = 89)  No (N = 86)  Chronic lung diseases  Yes (N = 50)  No (N = 125)  Malignant diseases  Yes (N = 16)  No (N = 159) | 20 (13 - 23)  22 (10.5 - 24)  20 (13.2 - 23.7)  20 (12 - 23)  20 (12.5 - 23)  20 (13 - 24)  20.5 (6.5 - 23.2)  20 (13.5 - 23.5)  22.5 (18 - 23)  20 (13 - 24) | .4  .77  .71  .83  .26 |
| Number of comorbid conditions:  <1 (N = 54)  2 (N = 52)  >3 (N = 69) | 20 (9.7 - 24)  19.5 (14 - 23.7)  21 (13 - 23) | .94 |
| Functional status:  Dependent (N = 52)  Independent (N = 123) | 19.5 (9.2- 22)  21 (13 - 24) | .21 |
| eGFR^b^, mL/min/1.73m^2^  > 50 (N = 91)  < 50 (N = 83) | 21 (15 - 24)  19 (5 - 23) | .02* |
| Infiltrates on chest radiograph:  Yes (N = 159)  No (N = 16) | 20 (13 - 23)  23.5 (17 - 25.5) | .13 |
| Hemolobin, g/dL:  > 10 (N = 147)  < 10 (N = 28 ) | 20 (14 - 24)  17.5 (0 - 23) | .1 |

Data are presented as median (IQR). ^a^ Number of days from admission to day 30 that the patient was not admitted to the hospital. ^b^Glomerular filtration rate estimated by CKD-EPI Creatinine Equation. *Statistically significant difference

**Table S3** Severity at admission and hospital-free days within 30 days of all patients hospitalized with respiratory syncytial virus-associated acute respiratory illness

| Severity at admission | Hospital-free days within 30 days^a^,  median (IQR), days | *P* value |
| --- | --- | --- |
| ICU admission:  Yes (N = 14)  No (N = 161) | 10.5 (0 - 14.2)  21 (15 - 24) | .002* |
| Invasive mechanical ventilation:  Yes (N = 36)  No (N = 139) | 8 (0 - 17.7)  22 (17 - 24) | <.001* |
| Vasopressor requirement:  Yes (N = 11)  No (N = 164) | 7 (0 - 19)  20.5 (13.2 - 24) | .006* |
| Minor criteria ≥3^b^:  Yes (N = 56)  No (N = 119) | 16 (5 - 21)  22 (17 - 24) | <.001* |
| Confusion/disorientation:  Yes (N = 22)  No (N = 153) | 12 (0 - 20.7)  21 (14 - 24) | .01* |
| Hypotension:  Yes (N = 7)  No (N = 168) | 15 (7 - 19)  20 (13 - 24) | .07 |
| Non-invasive ventilation:  Yes (N = 21)  No (N = 154) | 19 (13.5 - 22)  20 (12.7 - 24) | .33 |
| PaO_2_/FiO_2_ ratio <250 mmHg:  Yes (N = 93)  No (N = 82) | 20 (13 - 23)  20 (12.7 - 24) | .59 |
| Multilobar infiltrates:  Yes (N = 88)  No (N = 87) | 18 (5.2 - 22)  22 (18 - 25) | <.001* |
| Blood urea nitrogen level >20 mg/dL:  Yes (N = 75)  No (N = 100) | 18 (5 - 23)  21.5 (15.2 - 24) | .01* |
| WBC <4000 cells/mm^3^:  Yes (N = 10)  No (N = 165) | 14.5 (6.7- 19.2)  20 (13 - 24) | .07 |
| Platelet count <100,000 cells/mm^3^:  Yes (N = 14)  No (N = 161) | 17 (9.7 - 19.5)  20 (13 - 24) | .12 |
| Severe acute respiratory illness^c^:  Yes (N = 78)  No (N = 97) | 15 (0 - 20)  23 (20 - 24.5) | <.001* |

Data are presented as median (IQR). ^a^ Number of days from admission to day 30 that the patient was not admitted to the hospital.  ^b^IDSA/ATS minor criteria for severe community-acquired pneumonia [1]. ^c^Defined by IDSA/ATS criteria for severe community-acquired pneumonia [1]. *Statistically significant difference

**Table S4** Bacterial coinfection, bacterial superinfection, treatment and hospital free days within 30 days of all patients hospitalized with respiratory syncytial virus-associated acute respiratory illness

|  | Hospital-free days within 30 days^a^,  median (IQR), days | *P* value |
| --- | --- | --- |
| Positive blood culture  Yes (N = 3)  No (N = 132) | 4 (2 - 10.5)  20 (12 - 23) | .08 |
| Bacterial coinfection  Yes (N = 30)  No (N = 145) | 16 (3 - 20.2)  21 (14.5 - 24) | .002* |
| Bacterial superinfection  Yes (N = 18)  No (N = 157) | 0 (0 - 5.2)  21 (16.5 - 24) | <.001* |
| Bacterial infection^b^  Yes (N = 41)  No (N = 134) | 9 (0 - 19)  22 (17 - 24) | <.001* |
| Initial antibiotic treatment  Received (N = 150)  Not received (N = 25) | 20 (10.7 - 23)  22 (18 - 25) | .06 |
| Adequate initial antibiotic treatment^c^  Adequate (n = 23)  Not adequate (n = 7) | 17 (4 - 20)  9 (0 - 23) | .8 |
| Ribavirin treatment  Yes (N = 99)  No (N = 76) | 18 (9 - 22)  22.5 (17.2 - 25) | .001* |
| Bronchodilator therapy  Yes (N = 157)  No (N = 18) | 20 (12 - 23)  24 (22.7 - 27) | <.001* |
| Systemic corticosteroids use  Yes (N = 52)  No (N = 123) | 18.5 (1.2 - 23)  21 (14 - 24) | .03* |
| Non-respiratory nosocomial infections  Yes (N = 12)  No (N = 163) | 8 (0 - 12)  21 (15 - 24) | <.001* |

Data are presented as median (IQR). ^a^ Number of days from admission to day 30 that the patient was not admitted to the hospital. ^b^Bacterial coinfection and/or superinfection. ^c^Pathogens detected were susceptible to the antibiotics administered within 24 h of presentation. *Statistically significant difference

**References**

# 1. Metlay JP, Waterer GW, Long AC, et al. Diagnosis and Treatment of Adults with Community-acquired Pneumonia. An Official Clinical Practice Guideline of the American Thoracic Society and Infectious Diseases Society of America. *Am J Respir Crit Care Med*. 2019;200(7):e45-e67.
